# Identification and similarity analysis of aroma substances in main types of Fenghuang Dancong tea

**DOI:** 10.1371/journal.pone.0244224

**Published:** 2020-12-21

**Authors:** Zhangwei Li, Juhong Wang

**Affiliations:** 1 Institute of Chemistry and Environment Engineering, Hanshan Normal University, Chaozhou, P. R. China; 2 Institute of Food Engineering and Biotechnology, Hanshan Normal University, Chaozhou, P. R. China; ICAR - National Research Center on Plant Biotechnology, INDIA

## Abstract

Fenghuang Dancong tea covers the oolong tea category and is widely acknowledged for its unique floral and honey flavor. In order to characterize the volatile components in nine different aroma types of Fenghuang Dancong tea, the Headspace solid-phase microextraction (HS-SPME) coupled with gas chromatography-mass spectrometry (GC- MS) were employed. In addition, the similarity analysis and cluster analysis (CA) were performed to compare the aroma characteristics and establish the correlation between the nine types of teas. The principal component analysis (PCA) and orthogonal partial least squares discrimination analysis (OPLS-DA) method were employed to determine the volatile components with a high contribution to the overall aroma of each type of tea. The results presented a total of 122 volatile aroma components including 24 kinds of alcohol, 23 kinds of esters, 15 kinds of olefins, 12 kinds of aldehydes, 12 kinds of ketones, 13 kinds of alkanes and 23 kinds of other components from the nine types of Fenghuang Dancong tea. Of these volatile aroma components, 22 types were common with linalool, dehydrolinalool, linalool oxide I, linalool oxide II, etc. The similarity of the nine types of Fenghuang Dancong tea was found between 46.79% and 95.94%. The CA indicated that the nine types of Fenghuang Dancong tea could be clustered into four categories when the ordinate distance reached to 10. The PCA demonstrated that decane, octadecane, 2,2,4,6,6-pentamethylheptane, dehydrolinalool, geraniol and nerol were the important aroma components to Fenghuang Dancong Tea. OPLS-DA proved that 2,2,4,6,6-pentamethylheptane, dehydrolinalool, phenylacetaldehyde, nerolidol, linalool oxide I and hexanal were the key differential compounds between the various types of tea samples. This study provides a theoretical basis for characterizing the volatile aroma components in the main types of Fenghuang Dancong tea as well as the similarity and correlation between various types of Fenghuang Dancong tea.

## Introduction

Fenghuang Dancong tea is a very old tea which dates back to a period 700 years ago [1] and is cultivated on Fenghuang Mountain in the east of Guangdong Province, China. It belongs to oolong tea category and is well known for its unique floral and fruity aroma. It also serves as an important indicator in characterizing flavor and quality [2]. There are several types of Fenghuang Dancong tea exist; however, according to the sensory evaluation of tea aroma, the Fenghuang dancong tea can be traditionally divided into Youhua Xiang, Qilan Xiang, Huangzhi Xiang, Yelai Xiang, Xinren Xiang, Tongtian Xiang, Qunti Xiang, Zhilan Xiang, and Moli Xiang etc [3]. The aroma quality is an important factor that decides the flavor and price of Fenghuang Dancong tea. However, for a long time, the people’s perceptions have been determining the aroma characteristics of different types of Fenghuang Dancong tea, which are highly subjective and lack unified objective data support. Thus, few reports on the analysis and identification of the aroma characteristics of different types of Fenghuang Dancong tea exist currently [1]. In addition, researches on the similarity of tea aroma and the correlation between the main types of Fenghuang Dancong tea are very rare that have adversely affected the production and development of Fenghuang Dancong tea.

The relative content of tea aroma substances is low and complex. Therefore, GC-MS is often employed for the analysis and identification of aroma substances in tea [4, 5]. Further, the simultaneous distillation extraction (SDE) method is employed to extract the aroma components of tea as the extraction method is the basis of analysis and identification [6]. This method concurrently heats the sample and the extractant to mix their vapors at the fullest in a closed chamber and the extraction is repeated. It also maintains a prolonged high-temperature environment for the extraction that alters the aroma composition. Hence, the obtained extract cannot fully reflect the original aroma characteristics of the sample [7]. The headspace solid phase micro-extraction method (HS-SPME) equipped with a quartz glass fiber coated polymer as an adsorbent, is a solvent-free extraction method for aroma components that integrates sampling, extraction, concentration, and injection. This method allows for easy extraction and analysis of the samples at the inlet of the gas chromatography [8, 9]. The HS-SPME has the advantages of simple operation and mild conditions reflecting the original aroma of the tea to the greatest extent. This method is well adapted for the aroma extraction of black tea [10], green tea [11], pu’er tea [12] and oolong tea [13]. Ma [13] analyzed a type of oolong tea sample named “huangdan” for its volatile components producing from different processing steps by HS-SPME and GC-MS. The results confirmed that HS-SPME combined with GC-MS for assessing aroma compounds of oolong teas was accurate, sensitive and fast. Fenghuang dancong tea belongs to oolong tea. But until now, there are few reports involving of extracting volatile components of Fenghuang dancong tea by application of HS-SPME.

Therefore, in this study, the HS-SPME method was employed to extract volatile aroma components of tea, followed by the GC-MS combined method to analyze and identify the aroma components. In addition, the similarity analysis and CA were performed to compare the aroma characteristics of nine types of fenghuang dancong tea, and the volatile components contributing to the aroma in these types were identified by the PCA and OPLS-DA method. This study provided a quality assessment and comprehensive comparison of the volatile aroma of different types of fenghuang dancong tea. Attributing to this, our study also provides a certain theoretical basis for the selection, development, and utilization of fenghuang dancong tea.

## Materials and methods

### Tea samples

The fresh leaves of 9 types of Fenghuang Dancong tea were collected from Fenghuang Mountain, Chao’an County, Guangdong Province, China in April 2019 and were identified by three tea experts as Youhua Xiang, Qilan Xiang, Zhilan xiang, Yelai Xiang, Moli Xiang, Xinren Xiang, Huangzhi Xiang, Tongtian Xiang and Qunti Xiang. The tea samples were identified by three tea experts based on the appearance characteristics of tea leaves, as well as the information provided by the tea farmers who planted these tea trees. The tea samples were processed according to the local Fenghuang Dancong tea processing method [14]. The tea making process was as follows: after collecting the leaves, they were dried under the sun for 30 minutes, then kept at room temperature and stirred for 60 minutes. Later, the tea leaves were heated at 280° C for 30 minutes, and finally dried at 120° C for 100 minutes. The tea leaves were then grounded and passed through a 60-mesh sieve, sealed, and stored for testing.

### Extraction of aroma components by HS-SPME

10g each from the 9 kinds of tea leaves were weighed and transferred into a 150ml sample bottle, magnetically stirred after adding 30ml of ultrapure water, and finally seal with Teflon / silicone septa. The sample bottle was kept in 60° C water bath and balanced for 5min. Then, a manual sampler equipped with a 65-μm PDMS / DVB extraction head that has completed aging was inserted. After the headspace extraction at 60°C for 60 minutes, it was removed, immediately inserted into the inlet of the GC equipment for desorption for 5 minutes and the data was recorded [15].

### Analysis of aroma components by GC-MS

Gas chromatography equipped with HP-5MS flexible quartz capillary column (30 m × 0.25 μm × 0.25 μm) (Agilent HP5973N) was used. The inlet temperature was kept at 250°C. High purity helium (purity> 99.9999%) as a carrier gas, splitless injection was done and the flow rate was maintained at 1ml / min. The column temperature programming was as follow: the initial temperature was kept at 60°C with hold time of 5 min, ramped to 120°C at a rate of 3°C • min^-1^, held for 2min; ramped to 180°C at 5°C• min^-1^, held for 2min; finally ramped at a rate of 10°C • min^-1^ to 250°C and keep on. MS conditions: electron ionization source, the ion source temperature maintained at 230°C, electron energy 70eV, quadrupole temperature at 150°C, mass scanning range 40-400AMU.

Each volatile aroma compound was identified based on the data library of the National Institute of Standards and Technology (NIST98 MS) and compared their retention time indices (RIs) with the related references [16]. The RIs of aroma compounds were calculated according to the method of Song [17]. In order to obtain the RIs, a series of n-alkane (C_8_-C_25_, Anpel, China) was used under the same GC conditions.

Relative proportions of the components were determined from normalized FID peak areas, and the relative content was calculated by the following equation:

Relativecontent(%)=(singleconstituentarea/totalarea)×100%.

The aroma similarity was calculated according to the method of Xiao [18]. The SPSS 22.0 was used for the CA and ROC curves analysis. PCA and OPLS-DA were analysed by Simca 14.0.

## Results and discussion

### The result of volatile components of 9 types of Fenghuang Dancong tea analyzed by GC-MS

**Fig 1:**
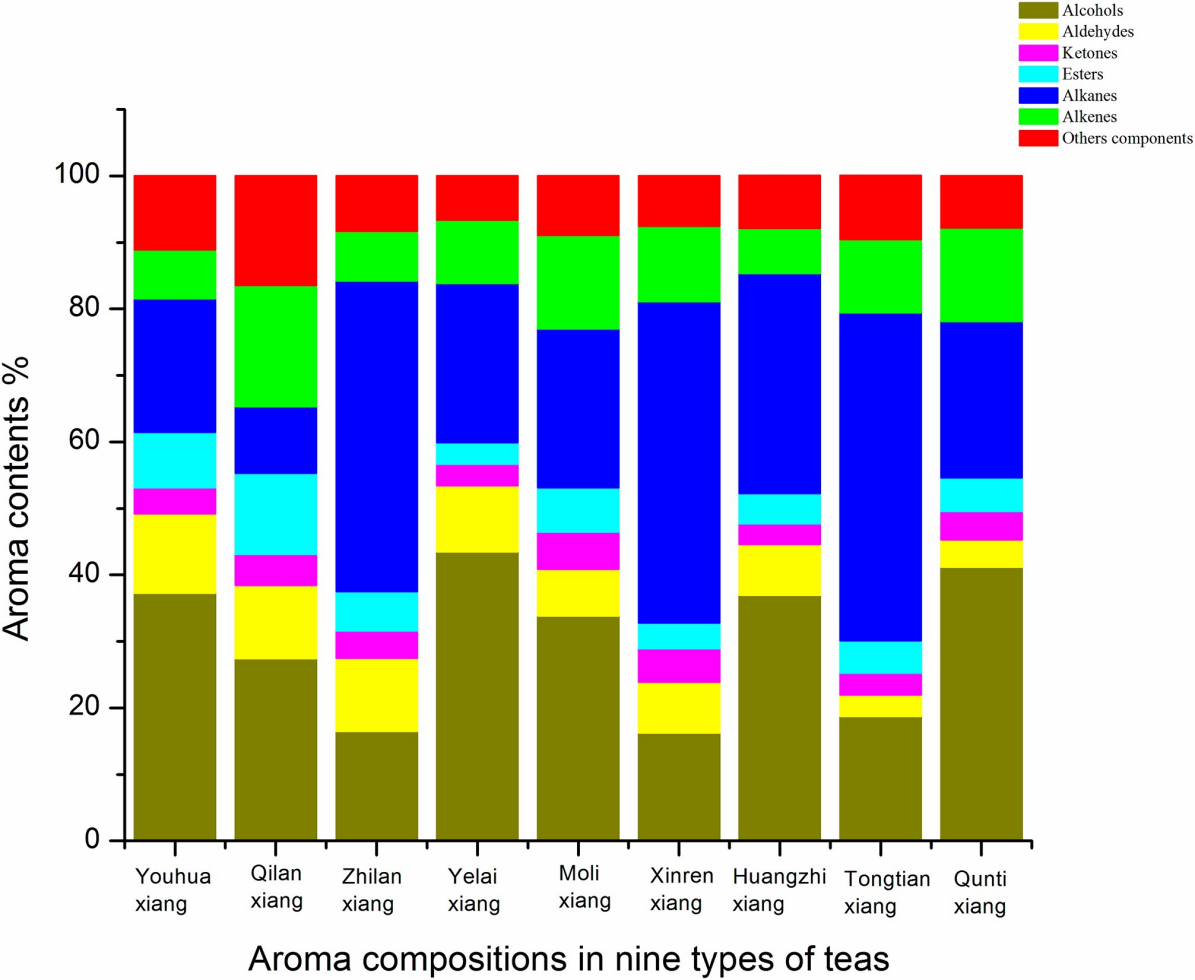
Different chemical classes of volatile compounds and their relative contents found in nine types of Fenghuang Dancong tea.

Table 1 demonstrated the result of volatile components of 9 types of Fenghuang Dancong tea analyzed by GC-MS. The GC-MS analysis detected a total of 122 kinds of volatile aroma components from 56 species of Youhua Xiang tea, 74 species of Qilan Xiang tea, 51 species of Zhilan Xiang tea, 63 species of Yelai Xiang tea, 77 species of Moli Xiang tea, 40 species of Xinren Xiang tea, 61 species of Huangzhi Xiang tea, 44 species of Tongtian Xiang tea, and 69 species of Qunti Xiang tea. Of these volatile aromas, there were 24 kinds of alcohol, 23 kinds of esters, 15 kinds of alkenes, 12 kinds of aldehydes, 12 kinds of ketones, 13 kinds of alkanes, 6 kinds of other compounds. A total of 22 types of volatile aromas were found in all samples with the highest contributing components such as linalool, dehydrolinalool, β-linalool oxide I, linalool oxide II, geraniol, nerolidol, phytol and 2,2, 4,6,6-pentamethylheptane, etc. The total contents of various components including alcohols, ketones, esters, aldehydes, alkanes, alkenes and other compounds in the volatile aromas of 9 types of Fenghuang Dancong tea are depicted in Fig 1.

**Table 1:** The results of volatile compounds of Fenghuang Dancong tea detected by GC-MS.

NO.	RI	Compounds	Relative content (%)								
			Youhua Xiang	Qilan Xiang	Zhilan Xiang	Yelai Xiang	Moli Xiang	Xinren Xiang	Huangzhi Xiang	Tongtian Xiang	Qunti Xiang
1	782	2-Pentene,4,4-dimethyl	-	-	-	-	-	-	-	4.27	2.61
2	802	Hexanal	4.61	2.14	8.01	5.18	2.79	5.38	4.02	-	-
3	811	2(1H)-Pyridinone	-	-	1.09	1.14	0.90	1.11	1.55	2.24	1.79
4	817	Pyrimidine,4-methyl	-	0.65	-	-	-	-	-	-	-
5	831	Furfural	-	1.14	-	-	-	-	-	-	-
6	848	Cyclopentanone,2-methy	1.27	0.43	0.51	-	0.61	-	-	-	-
7	859	Ethylbenzene	1.82	0.82	1.75	0.98	0.84	1.65	1.36	2.18	0.78
8	893	O-xylene	6.72	4.34	5.53	2.82	2.88	5.47	4.03	6.62	2.27
9	902	2-Heptanone	-	-	0.66	0.40	-	0.47	-	-	-
10	910	Pyrimidine,4,6-dimethyl	-	1.37	-	-	-	-	-	-	-
11	918	1,4-Benzenediamine	-	0.36	-	-	-	-	-	-	-
12	957	Benzaldehyde	1.95	3.39	0.63	1.10	1.16	0.73	0.61	0.84	1.32
13	968	Hexanoic acid	-	-	-	-	0.21	-	-	-	-
14	981	Methyl 2-furoate	-	1.75	-	-	-	-	-	-	-
15	985	Dodecanal	-	-	-	0.30	-	-	-	-	-
16	989	β-Eudesmol	-	0.87	0.80	0.69	0.69	1.25	0.40		0.40
17	992	2,2,4,6,6-Pentamethylheptane	6.26	3.91	19.49	9.47	10.21	20.75	12.99	20.32	9.99
18	995	9-Tetradecane-1-ol	-	-	0.49	-	-	0.55	-	-	-
19	1001	Decane	1.35	0.80	3.38	1.51	1.85	3.96	2.29	3.95	1.51
20	1004	Heptane-2,3-dione, 1,7,7-trimethyl	-	-	0.72	0.46	0.56	1.43	-	-	-
21	1010	Octanal	-	-	-	-	-	0.26	-	-	-
22	1012	2-Ethyl-5-methylpyrazine	-	-	0.60	0.50	0.31	0.40	-	-	-
23	1014	α-Terpinene	-	0.85	-	-	-	-	-	-	-
24	1022	O-cymene	-	-	0.65	0.62	0.33	0.87	0.39	0.49	0.49
25	1030	Limonene	1.90	1.39	3.15	1.93	2.10	4.95	1.64	2.54	1.79
26	1034	Cis-β-ocimene	1.40	1.35	2.98	2.31	2.09	5.93	1.15	1.48	1.33
27	1040	Benzylalcohol	0.82	0.66	1.26	1.08	0.89	1.51	0.78	0.85	0.72
28	1047	Trans-2-decen-1-ol	-	-	-	-	-	-	0.30	-	-
29	1051	Phenylacetaldehyde	1.36	1.15	1.15	1.24	1.49	1.33	1.49	1.16	1.30
30	1057	γ—Terpinene	1.85	8.47	0.76	2.21	2.02	-	1.57	0.78	2.65
31	1064	Isophorone	-	-	0.71	0.29	-	0.61	-	0.46	0.20
32	1073	1-Octanol	-	-	-	-	0.49	-	0.43	-	0.17
33	1074	4-Hydroxybenzylalcohol	0.87	1.36	0.43	-	-	-	-	-	0.24
34	1078	Linalool oxide I	5.79	5.30	1.92	6.54	5.62	1.11	2.74	1.27	4.36
35	1084	2,3-Diethylpyrazine	-	0.55	-	-	-	-	-	-	-
36	1094	Linalool oxide II	4.20	3.22	1.25	3.58	3.65	1.63	1.78	1.03	2.86
37	1098	Nonanal	2.59	1.44	1.21	1.04	0.75	-	1.01	1.21	0.53
38	1101	Undecane	5.04	2.09	11.45	5.57	6.05	14.02	8.59	14.32	5.95
39	1104	Linalool	11.19	2.12	3.41	13.98	5.82	1.71	8.71	2.29	4.33
40	1108	Dehydrolinalool	9.22	1.24	3.57	13.92	4.62	1.10	15.14	3.59	10.16
41	1117	2-Undecanone	-	0.46	-	-	-	-	-	-	0.14
42	1117	Dodecanal	0.28	-	-	-	-	-	-	-	-
43	1120	α-Cyclocitral	0.30	1.06	-	0.45	0.50	-	0.50	-	0.59
44	1135	2-Methylbenzonitrile	-		-	0.31	0.22	-	0.31	-	-
45	1147	Benzonitrile	-	1.41	0.39	1.66	1.24	-	0.74	0.49	0.90
46	1151	4-Isopropylcyclohexanone	-	-	-	0.26	0.22	-	-	-	-
47	1162	1-Nonanol	-	0.64	-	-	0.39	-	-	-	0.59
48	1173	Naphthalene	-	0.68	-	-	-	-	-	-	-
49	1177	Epoxylinalool	-	0.66	-	-	0.22	-	-	-	0.38
50	1187	Cis-3-Hexenyl butyrate	-	-	-	0.46	-	-	-	-	-
51	1193	3,4-Dimethyl-o-phenylenediamine	-	2.32	-	-	0.69	-	-	-	0.35
52	1200	Dodecane	2.96	1.26	3.76	3.16	2.40	3.30	3.60	3.95	2.31
53	1205	Methyl Salicylate	0.32	1.04	-	0.47	0.49	-	-	-	-
54	1208	Decanal	0.90	0.40	-	0.42	0.33	-	-	-	0.38
55	1225	P-cyclocitral	-	-	-	0.23	-	-	-	-	-
56	1231	Hexyl pivalate	0.49	0.35	-	0.31	0.26	-	-	-	-
57	1235	Nerol	-	-	-	0.32	-	-	0.31	-	0.37
58	1240	Hept-2-ene, 3,7,7-trimethyl				0.24	-	-	-	-	-
59	1260	Geraniol	1.86	1.81	0.57	1.78	2.21	0.58	1.97	0.81	2.21
60	1271	Nonanoic acid	-	0.54	-	-	0.25	-	-	-	-
61	1293	Pentyl hexanoate	0.48	0.52	-	0.25	0.57	-	-	-	0.49
62	1300	Tridecane	-	-	-	-	-	-	-	0.28	-
63	1306	Indol	-	0.39	-	0.34	0.50	-	0.50	0.33	0.59
64	1310	2-Methyl naphthalene	-	-	-	-	0.22	-	-	-	0.44
65	1327	Methyl geranate	-	0.56	-	-	0.61	-	0.39	-	0.50
66	1351	α-Ionene	-	-	-	-	0.25	-	0.35	-	-
67	1359	Hydroxyundecanolide	-	0.55	-	-	-	-	-	-	-
68	1368	2-Naphthalene ethanol	-	0.89	-	-	0.33	-	-	-	-
69	1372	(E)-2-Hexenyl hexanoate	-	-	0.44	0.24	0.25	-	-	0.43	-
70	1385	(Z)-3-Hexenyl hexanoate	-	1.13	-	-	0.41	-	0.30	-	-
71	1388	Hexyl hexanoate	-	0.53	-	-	-	-	-	-	-
72	1391	Cis-jasmone	0.61	0.78	-	0.27	1.01	-	0.58	-	0.45
73	1401	Tetradecane	1.53	0.77	3.60	1.63	1.43	2.75	2.19	3.12	1.16
74	1410	α-Cedrene	-	-	-	1.55	2.34	-	0.97	0.99	2.15
75	1439	(E)-P-Famesene	0.34	0.56	-	-	0.49	-	-	-	-
76	1444	Geranylacetone	0.56	0.85	-	0.27	0.91	-	0.30	-	0.37
77	1455	(E)-Geranyl acetone	-	-	-	-	0.28	-	-	-	0.40
78	1459	(E)-β-Farnesene	0.47	0.83	-	0.26	0.71	-	0.39	-	0.40
79	1465	γ-Cadinene	0.26	-	0.58	0.28	-	0.44	0.36	0.50	-
80	1498	β-Bisabolene	1.10	2.68	-	0.48	2.07	-	0.35	-	1.85
81	1501	Pentadecane	0.29	-	0.89	0.29	0.25	-	-	-	-
82	1506	3,5-Di-tert-butylphenol	-	-	-	-	0.41	-	-	-	-
83	1514	Butylated hydroxytoluene	-	-	-	-	-	-	0.49	-	1.11
84	1520	2,4-Bis (1,1-dimethylethyl) phenol	-	-	-	-	0.26	-	-	-	-
85	1531	Dihydroactinidiolide	-	0.22	-	-	-	-	-	-	-
86	1538	Methyl anthranilate	-	0.58	-	-	0.71	-	-	-	0.48
87	1565	E-3,7,11-trimethyl 1,3,6,10-dodecatrien-3-ol	-	-	0.40	-	-	-	-	-	-
88	1572	Nerolidol	2.08	7.10	1.24	1.28	8.04	5.47	2.98	7.59	12.99
89	1583	1-Hexadecene	-	1.20	-	0.24	1.23	-	-	-	0.56
90	1588	(Z)-3-Hexen-1-ol,benzoate	-	-	-	-	0.28	-	-	-	-
91	1594	Cis-7-tetradecene aldehyde	-	0.34	-	-	-	-	-	-	-
92	1597	1-Tridecane	-	0.32	-	-	0.39	-	-	-	0.34
93	1601	Hexadecane	1.35	0.65	1.89	0.86	0.80	1.65	1.28	1.64	0.81
94	1627	Cadinol1,4-diene-3-ol	-	-	-	-	0.39	-	-	-	-
95	1658	Tributyl phosphate	3.97	1.48	0.50	0.34	0.41	0.72	0.77	0.88	0.30
96	1660	α-Cadinol	-	-	-	-	-	-	-	-	0.34
97	1667	Decylcyclohexane	-	-	0.49	-	-	-	0.33	-	0.29
98	1676	Cadalene	-	0.59	-	-	0.39	-	-	0.43	0.43
99	1700	Heptadecane	0.46	0.56	0.56	0.36	0.27	0.53	0.54	0.73	0.35
100	1710	2-Hexyl-l-decanol	0.27	0.35	0.43	-	-	-	-	-	-
101	1745	Phenanthrene	-	-	-	-	-	-	0.48	-	-
102	1800	Octadecane	0.56	-	0.59	0.27	**0.29**	0.54	**0.47**	0.58	**0.39**
103	1811	Ethylene glycol monolauryl ether	-	-	0.82	0.37	0.36	-	0.56	0.76	0.51
104	1843	Methyl pentadecanoate	-	0.46	-	-	0.51	-	-	-	0.45
105	1850	2-Terecanone	1.15	2.12	-	-	1.10	1.39	0.38	0.63	0.67
106	1864	Eicosane	0.32	-	-	0.21	-	-	0.43	-	0.27
107	1868	(Z)-9-Tetradecen-l-ol acetate	0.23	-	-	0.18	0.31	-	0.33	-	0.36
108	1901	Famesyl acetone	0.30	-	0.42	0.17	-	-	0.33	-	0.28
109	1930	Methylhexadecanoate	0.68	1.97	-	-	1.28	1.36	0.47	0.75	1.11
110	1953	Isophytol	0.35	0.55	-	-	-	-	-	-	-
111	1965	Palmitic acid	0.33	-	-	-	-	-	-	-	-
112	1966	Hexadecylic acid	-	0.74	-	-	-	-	-	-	-
113	1976	Didodecyl Phthalate	0.28	-	0.40	-	-	-	-	-	-
114	2001	Ethyl palmitate	0.34	-	0.53	0.22	-	0.43	0.37	0.47	0.36
115	2039	Gerany linalool	-	-	-	-	-	-	0.39	-	0.42
116	2064	Methyl linoleate	0.39	-	0.66	0.29	0.24	0.48	0.53	0.61	0.49
117	2073	Methyl oleate	0.40	-	0.90	-	-	-	0.36	0.39	-
118	2101	Phytol	0.61	0.67	0.76	0.30	0.55	0.75	0.58	0.72	0.68
119	2109	Methyl octadecanoate	0.39	1.54	-	-	0.47	-	-	-	0.38
120	2120	Linoleic acid	2.20	1.77	-	-	0.34	-	-	-	0.88
121	2201	Octadecyl acetate	0.38	-	1.62	0.15	-	0.85	0.43	0.53	-
122	2211	1-Tetradecanol	-	-	-	-	-	0.56	0.45	0.59	-

Of the common aroma components, β-linalool, dehydrolinalool, linalool oxide I, linalool oxide II, geraniol, nerol, and phytol exhibited higher aroma content. Terpene synthase catalyzation produces β-Linalool and geraniol from geranyl pyrophosphate, and β-linalool further produces derivatives such as dehydrolinalool, linalool oxide I and linalool oxide II [19]. β-Linalool has a citrus-like aroma with a low threshold. Therefore, it can be sensed by the human sensory organs when the concentration is higher than 0.6μg / L [20]. β-Linalool has been detected in various aroma components of oolong tea. For example, Guo [21] reported that the content of β-linalool in oolong tea drawn down during the manufacturing process, suggesting it’s application in the preparation of tea. Linalool oxides I and II exhibit a sweet floral and fruity fragrance whereas geraniol exhibits rose fragrance and dehydrolinalool a spicy and lavender-like fragrance [22]. These add value to the quality and total aroma of fenghang dancong tea. Among the nine types of Fenghuang Dancong tea, alcohol accounts for 16.2–43.5% of the total aroma components. The Yelai xiang tea exhibited the highest proportion while Xinren Xiang tea showed the lowest proportion. It is believed that alcohols are the main aroma components of Fenghuang Dancong tea. Chen [23] has reported that in several types of oolong teas, the proportion of alcoholic aroma content ranges between 7.2–38.5%, which differs from black tea, green tea, and pu’er tea to a certain extent. Our results were consistent with the above study results. This is one of the reasons why the aroma of Fenghuang Dancong tea is different from other teas.

In addition, benzyl alcohol and benzaldehyde are the common aroma components common of Fenghuang Dancong tea/oolong tea [24]. Both the benzyl alcohol and benzaldehyde are produced by cinnamic acid under the catalysis of phenylalanine with related biological enzymes [25]. Benzyl alcohol exerts a sweet and fruity flavor, while benzaldehyde has a special almond smell [20]. Nerolidol has a sweet orange blossom flavor, slightly woody, and is an important aroma component in oolong tea. It is mainly produced by mevalonate in the cytosol of tea cells under the action of related biological enzymes [26]. Ma [24] demonstrated that the distribution of nerolidol contents in fresh tea leave is low and is mainly produced during the fermentation stage of tea. In contrast, our research revealed that the nerolidol contents in the nine types of Fenghuang Dancong tea are relatively high, ranging from 1.24% to 12.99%. Among the nine types of tea, Zhilan Xiang exhibited the lowest nerolidol content while the Qunti Xiang had the highest level. The Moli Xiang tea, Tongtian Xiang Tea and Qilan Xiang also had a higher level of nerolidol reaching to 8.04%, 7.59% and 7.10%, respectively.

Indole is an aroma substance of oolong tea in tea produced from the decomposition of tryptophan. At high concentrations, it exhibits an irritating odor and mild floral scent at low concentrations [27]. Phenylacetonitrile has a pungent odor and is mainly produced by phenylalanine in tea leaf cells [24]. It is also detected in the nine types of tea and is also an important component of the aroma in Fenghuang Dancong tea. Hexanal has the smell of green grass, giving the tea a fresh and elegant taste [28]. Among the nine types of tea samples, hexanal showed the highest contents in Zhilang Xiang tea (8.01%), Xinren Xiang tea (5.38%), Yelai Xiang tea (5.18%), and Youhua Xiang tea (4.61%). 2,2,4,6,6-pentamethylheptane has an irritating odor, which is often detected in some aroma components of oolong tea [23]. In this study, the content of 2,2,4,6,6-pentamethylheptane was between 2.30% -20.75%, which revealed that this compound had a greater contribution to the aroma of Fenghuang Dancong tea. Some monoterpenes and sesquiterpenes such as limonene, basilene, γ-terpinene, δ-juniperene, and farnesene, contribute significantly to the aroma of some tea types. Previous studies have found that monoterpenes and sesquiterpenes exhibit rich sweet, floral or woody aromas and their composition and relative content may attribute to the different aroma characteristics of Fenghuang Dancong tea [29]. For example, the content of limonene was found to be 4.95%, 3.15% and 2.54% in Xinren Xiang, Zhilan Xiang, and Tongtian Xiang tea, respectively. Limonene has a lemon fresh fragrance, giving the tea a light lemon fragrance. It is reported that limonene is an important aroma component and also often detected in green tea [30]. Additionally, the limonene content is high in higher grade of green tea [31]. Ocimene exerts a sweet fragrance and is relatively high in Xinren Xiang, Zhilan Xiang, and Yelai Xiang tea contributing to the aroma of these teas. γ-terpinene has citrus and lemon aromas [32]and the highest content of 8.47% was found in Qilan Xiang tea, indicating that γ-terpinene has a greater contribution to the aroma components of Qilanxiang tea. Among the nine types of tea, the volatile ester components mainly included ethyl palmitate, methyl linoleate, and methyl oleate. These esters are formed by the dehydration and condensation of some higher fatty acids and lower alcohols. These compounds exert poor volatility, milder flavor, and contribute less to the aroma of tea [33].

### The similarity analysis and CA on nine types of Fenghuang Dancong tea

**Fig 2:**
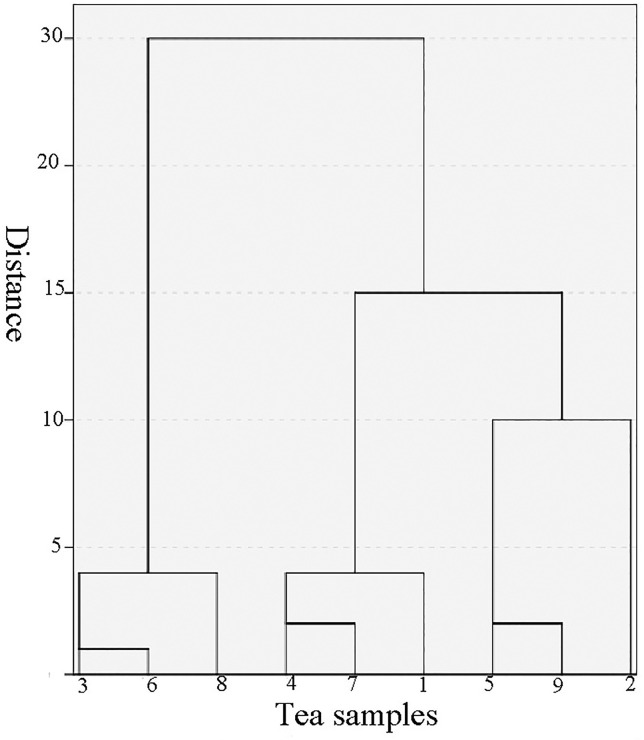
Cluster analysis of nine types of Fenghuang Dancong tea. 1-Youhua xiang, 2-Qilan xiang, 3-Zhilan xiang, 4-Yelai xiang, 5-Moli xiang, 6-Xinren xiang, 7-Huangzhi xiang, 8-Tongtian xiang, 9-Qunti xiang.

The similarity analysis provided a comprehensive comparison of the volatile aroma and established a relationship between different types of tea. This method provided a theoretical basis for the study of the internal connections between different types of tea [1]. The study results revealed a similarity ranging from 46.79% to 95.94% between the volatile aromas of the nine types of teas (Table 2). The similarity between Qilan Xiang and Zhilan Xiang tea showed the lowest range while the Zhilan Xiang and Xinren Xiang showed the highest similarity. The similarity between Youhua Xiang and Yelai Xiang tea, Zhilan Xiang and Xinren Xiang, Yelai Xiang and Huangzhi Xiang, Moli Xiang and Qunti Xiang, Xinren Xiang and Tongtian Xiang, Tongtian Xiang and Zhilan Xiang exhibited more than 90% similarity, revealing higher similarity between these types. The similarity between Zhilan Xiang and Qilan Xiang, Zhilan Xiang and Xinren Xiang, Xinren Xiang and Qilan Xiang tea showed 50% lower similarity. Cluster analysis is a multivariate statistical analysis that gradually aggregates samples according to the similarity of their quality characteristics and is widely used for the assessment of volatile components of tea [34]. Wang [35] analyzed the similarity between oolong tea types which revealed that the contents of volatile components in oolong tea types from the same area exhibited the most similarity and could be classified into the same category. It also demonstrated that the application of the cluster analysis method adds value to the classification of tea origin. In this study, the content of the volatile components of the nine types of Fenghuang Dancong tea were considered as a variable, and the euclidean distance square was considered as a metric to conduct the systematic cluster analysis. The analytical results showed that the nine types of Fenghuang Dancong tea can be grouped into 4 categories when the ordinate distance is 10 (Fig 2). The categorises are as follows: (1) Zhilan Xiang, Xinren Xiang and Tongtian Xiang tea; (2) Yelai Xiang, Huangzhi Xiang and Youhua Xiang tea; (3) Moli Xiang and Qunti Xiang tea; (4) Qilan Xiang tea as one category alone. These results revealed that Qilan Xiang tea exhibited less similarity to other types of tea, which is consistent with the results of the similarity analysis.

**Table 2:** Comparison of aromatics similarity analysis between nine types of Fenghuang Dancong tea (%).

Type	Youhua xiang	Qilan xiang	Zhilan xiang	Yelai xiang	Moli xiang	Xinren xiang	Huangzhi xiang	Tongtian xiang	Qunti xiang
Youhua xiang	-	63.46	68.09	92.64	81.47	58.91	87.19	60.90	72.34
Qilan xiang	63.46	-	46.79	51.55	76.00	48.87	50.07	50.91	69.69
Zhilan xiang	68.09	46.79	-	69.22	79.29	95.94	81.26	90.69	65.03
Yelai xiang	92.64	51.55	69.22	-	81.39	58.68	93.54	60.37	74.96
Moli xiang	81.47	76.00	79.29	81.39	-	80.30	84.11	82.13	91.72
Xinren xiang	58.91	48.87	95.94	58.68	80.30	-	73.59	94.11	68.15
Huangzhi xiang	87.19	50.07	81.26	93.54	84.11	73.59	-	77.76	82.92
Tongtian xiang	60.90	50.91	90.69	60.37	82.13	94.11	77.76	-	78.22
Qunti xiang	72.34	69.69	65.03	74.96	91.72	68.15	82.92	78.22	-
Average	73.12	57.17	74.54	72.79	82.05	72.32	78.81	74.39	75.38

### The PCA, OPLS-DA and ROC curves of nine types of Fenghuang Dancong tea

**Fig 3:**
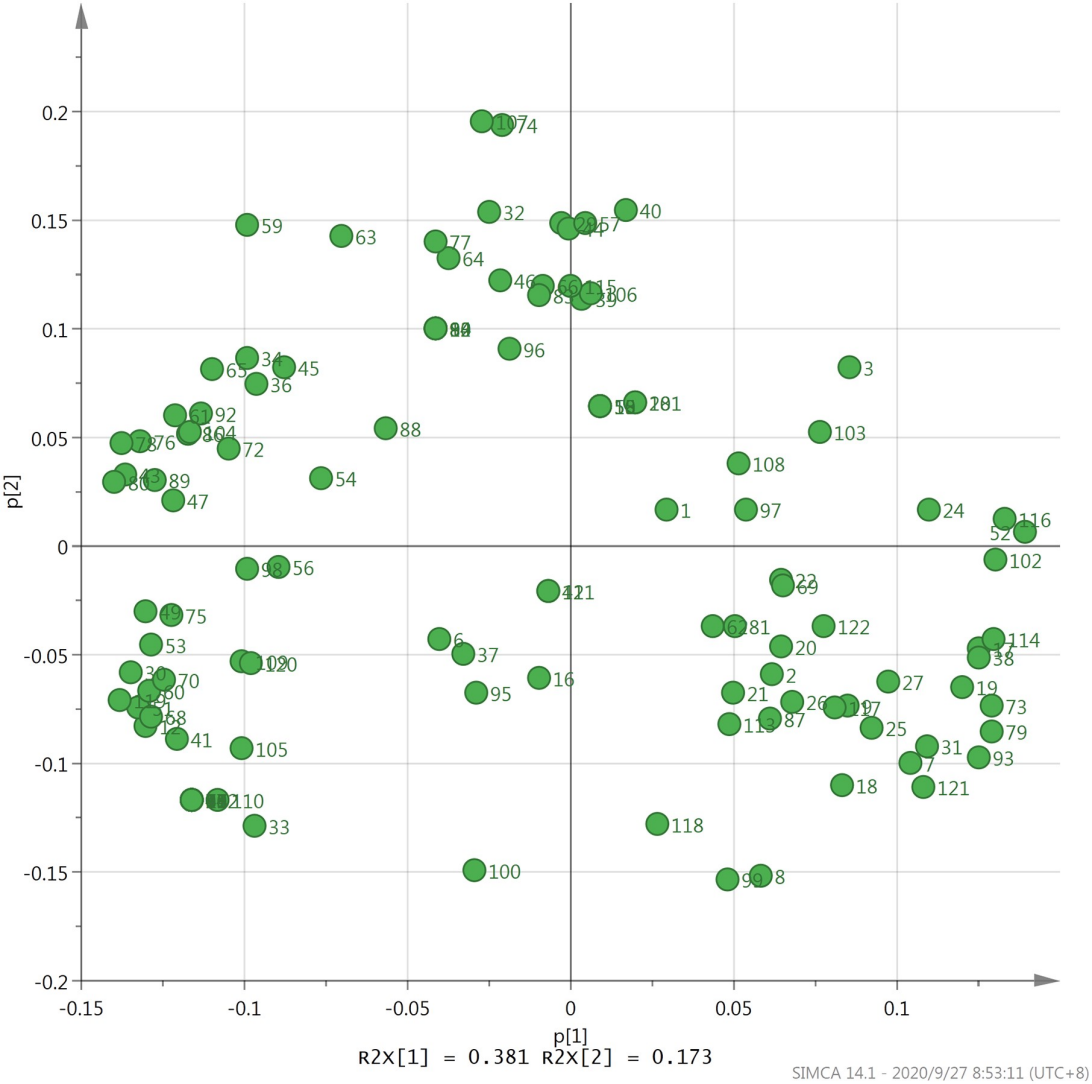
Principal component analysis results of the 122 kinds of common aroma compounds in 9 types of Fenghuang Dancong tea. Compound NO. was the same to Table 1.

PCA is a statistical method that converts multiple variables into a few comprehensive variables through dimensional reduction, thereby simplifying the data and interpreting most of the original information. This method is often used to explore the comprehensive variables between samples and explain the information among them [13, 36]. PCA has been widely used in the study of volatile components of tea [35]. In this study, the contents of 122 kinds of volatile components obtained from the GC-MS analysis of the 9 types of Fenghuang Dancong tea were applying for principal component analysis. The principal component characteristic value, contribution rate and cumulative contribution rate were depicted in Table 3. The cumulative variance contribution rate of the first five principal components showed 84.31%, indicating that the first five principal components were enough to explain all the variable information of the fragrance in Fenghuang Dancong tea. The first and second principal components showed 38.06% and 17.33% contribution rate, respectively. The results demonstrated that the volatile components which contributed more to PC1 were alkanes, including decane, octadecane, 2,2,4,6,6-pentamethylheptane, undecane, tetradecane and,hexadecane, etc. Volatile components with more floral and fruity fragrances contributing more to PC2 were alcohols which contained dehydrolinalool, geraniol, nerol and 1-octanol, etc. These results proved that decane, octadecane, 2,2,4,6,6-pentamethylheptane, undecane, tetradecane, hexadecane, geraniol, dehydrolinalool, nerol and 1-octanol were the important aroma-contributing ingredients of Fenghuang Dancong tea (Fig 3). These components played an important role in the volatile components of tea.

**Table 3:** Eigenvalues and variance contribution rates of principal components.

PC No.	Characteristic value	Contribution rate%	Cumulative%
1	46.43	38.06	38.06
2	21.15	17.33	55.39
3	14.27	11.70	67.09
4	11.44	9.38	76.47
5	9.57	7.84	84.31
6	6.72	5.51	89.82
7	6.50	5.33	95.15
8	5.92	4.85	100.00

**Fig 4:**
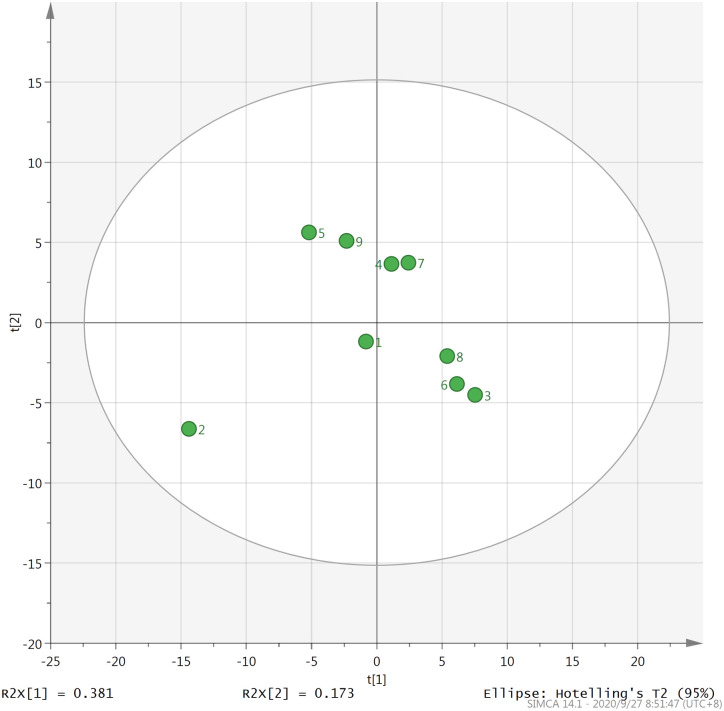
Principal component analysis of nine types of Fenghuang Dancong tea on PC1 and PC2. 1-Youhua xiang, 2-Qilan xiang, 3-Zhilan xiang, 4-Yelai xiang, 5-Moli xiang, 6-Xinren xiang, 7-Huangzhi xiang, 8-Tongtian xiang, 9-Qunti xiang.

PC1 and PC2 are the two main components contributing the most to the overall aroma. The score scatter plot of nine types of Fenghuang Dancong tea on PC1 and PC2 is depicted in Fig 4. As illustrated in the figure, the nine types of Fenghuang Dancong tea can be roughly divided into four categories on PC1 and PC2. The Yelai Xiang, Huangzhi Xiang, Moli Xiang and Qunti Xiang tea are relatively concentrated; hence, they can be classified into one category while Zhilan Xiang, Xinren Xiang and Tongtian Xiang tea belong to the same category. Youhua Xiang and Qilan Xiang tea is far away from other types and can be classified into one category respectively. The principal component analysis can effectively determine the components that have a greater contribution to the volatile aroma of tea. The results of the principal component analysis of 122 aroma components demonstrated that alkanes and alcohols exhibited the greatest influence on the aroma components of the nine types of Fenghuang Dancong tea. Dehydrolinalool, geraniol, nerol and 1-octanol exhibited rich floral and fruity aromas which are the main source of floral and fruity aromas of Fenghuang Dancong Tea and play an important role in forming the characteristic aroma of Fenghuang Dancong Tea. From the main component analysis of the volatile aroma components of tea, we can grasp the main key components affecting the aroma of tea and provide important evidence for studying the aroma characteristics of tea.

**Fig 5:**
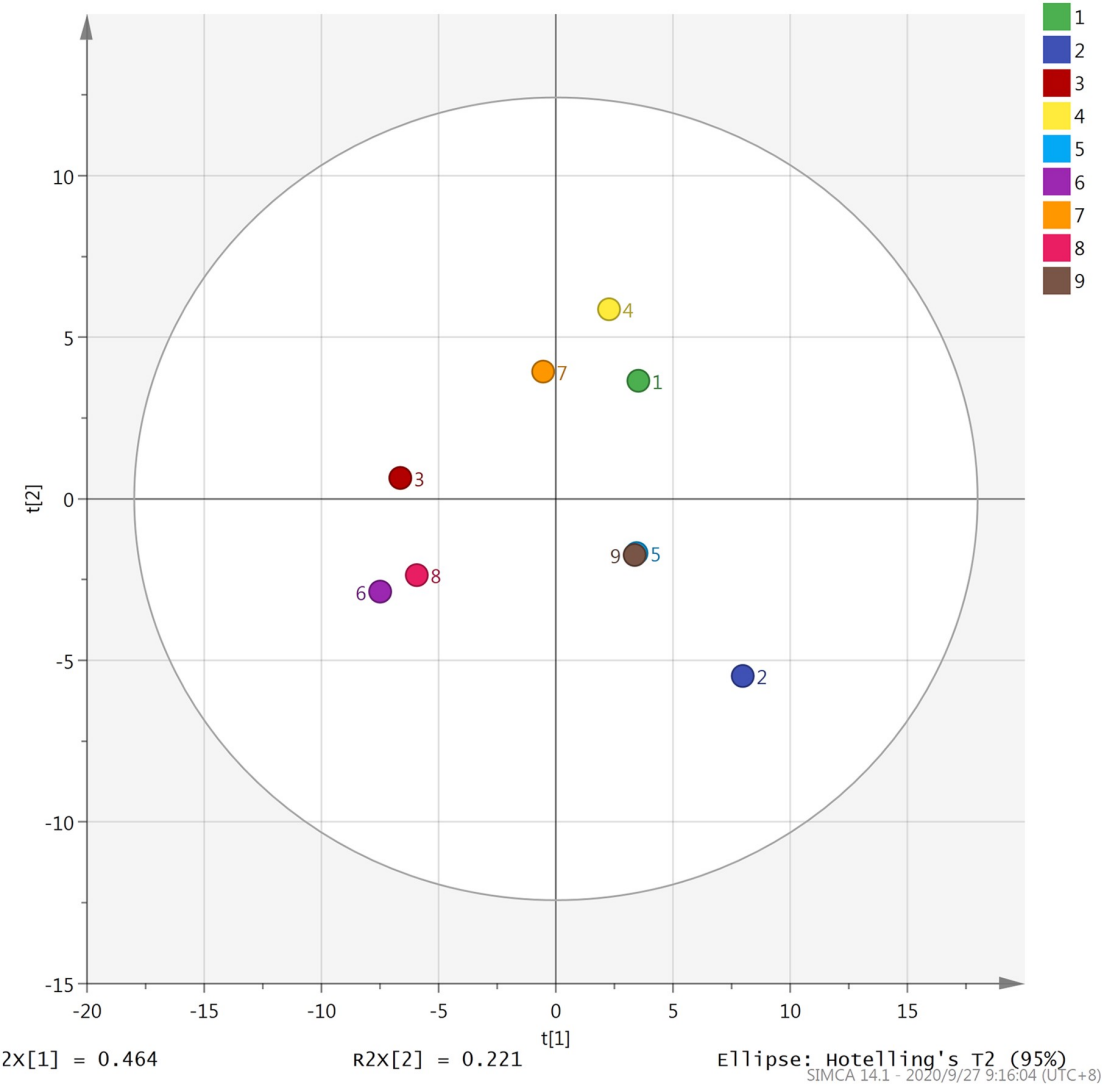
Orthogonal projections to latent structures data analysis of nine types of Fenghuang Dancong tea on PC1 and PC2. 1-Youhua xiang, 2-Qilan xiang, 3-Zhilan xiang, 4-Yelai xiang, 5-Moli xiang, 6-Xinren xiang, 7-Huangzhi xiang, 8-Tongtian xiang, 9-Qunti xiang.

**Fig 6:**
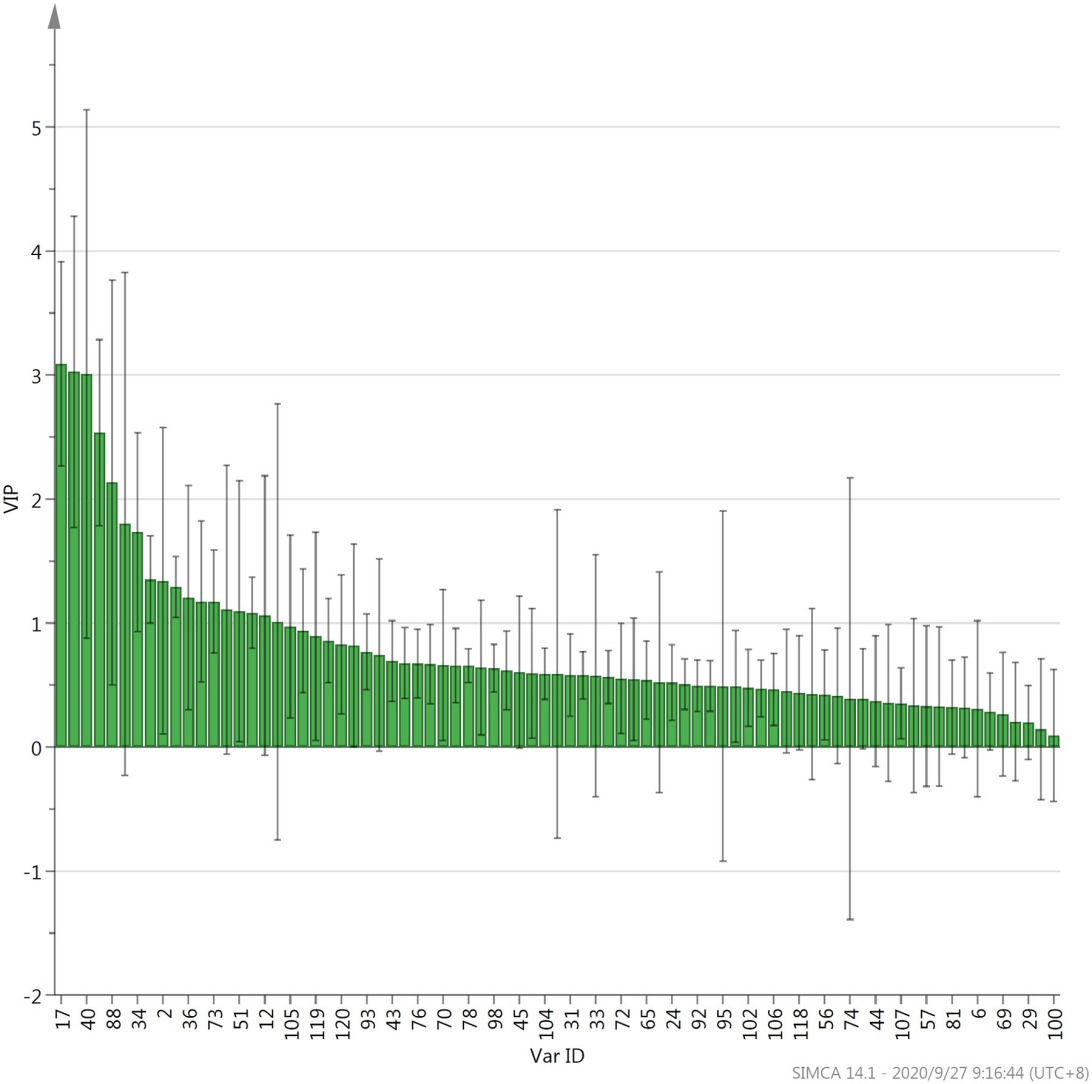
VIP values of nine types of Fenghuang Dancong tea. Compound NO. was the same to Table 1.

Orthogonal partial least squares discriminant analysis (OPLS-DA) is widely used in the analysis of metabolome data. Since this analysis method can maximize the difference between groups data, it is used to screen differential metabolites [37]. Fig 5 revealed that with the application of OPLS-DA method, the nine types of Fenghuang Dancong tea can be divided into four categories. The Zhilan Xiang, Xinren Xiang and Tongtian Xiang tea can be classified into one category. The Moli Xiang and Qunti Xiang tea belong to the same category while the other three tea types, Youhua Xiang, Yelai Xiang and Tongtian Xiang are the same category. Similar to the result of PCA, Qilang Xiang is far away from other types and can be classified into one category alone. Variable importance in project (VIP) in OPLS-DA model can indicate the key differential metabolites among the samples. In this experiment, the aroma compounds which VIP value ≥ 1 were considered to be the key differential volatile aroma between the nine types of Fenghuang Dancong tea. Result presented that VIP values of 2,2,4,6,6-pentamethylheptane, dehydrolinalool, phenylacetaldehyde, nerolidol, linalool oxide I, hexanal, linalool oxide II,tetradecane, 3,4-dimethyl-o-phenylenediamine and benzaldehyde were all ≥ 1, suggesting these volatile aromas were the key differential compounds between the various types of tea samples (Fig 6).

Receiver operating characteristic curve (ROC) is often used to reflect the performance of the classifier. Area under the curve (AUC) is an important criterion for measuring the quality of the classification model. The AUC value is generally between 0.5 and 1.0, and the larger the value, the better of the classification model [38]. Result presented that 2-methy cyclopentanone, o-xylene, linalool oxide II, nonanal decanal, hexyl pivalate, tributyl phosphate, palmitic acid and linoleic acid appeared to be the highest AUC value (AUC = 1.000) among the 122 kinds of tea components, suggesting that these volatile aroma components were suitable to be used for classification model for nine types of tea (Table 4).

**Table 4:** The AUC values of 122 kinds of aroma compounds.

Compound No.	AUC values	Compound No.	AUC values	Compound No.	AUC values	Compound No.	AUC values
1	0.375	32	0.313	63	0.125	94	0.438
2	0.625	33	0.875	64	0.375	95	1.000
3	0.063	34	0.875	65	0.250	96	0.438
4	0.438	35	0.438	66	0.375	97	0.313
5	0.438	36	1.000	67	0.438	98	0.250
6	1.000	37	1.000	68	0.375	99	0.375
7	0.875	38	0.125	69	0.250	100	0.750
8	1.000	39	0.875	70	0.313	101	0.438
9	0.313	40	0.625	71	0.438	102	0.750
10	0.438	41	0.375	72	0.750	103	0.125
11	0.438	42	1.000	73	0.375	104	0.313
12	0.875	43	0.375	74	0.188	105	0.750
13	0.438	44	0.313	75	0.750	106	0.875
14	0.438	45	0.063	76	0.750	107	0.625
15	0.438	46	0.375	77	0.375	108	0.750
16	0.063	47	0.313	78	0.750	109	0.375
17	0.125	48	0.438	79	0.375	110	0.875
18	0.375	49	0.313	80	0.625	111	1.000
19	0.125	50	0.438	81	0.813	112	0.438
20	0.250	51	0.313	82	0.438	113	0.875
21	0.438	52	0.375	83	0.375	114	0.375
22	0.250	53	0.625	84	0.438	115	0.375
23	0.438	54	1.000	85	0.438	116	0.375
24	0.063	55	0.438	86	0.313	117	0.875
25	0.375	56	1.000	87	0.438	118	0.375
26	0.375	57	0.313	88	0.250	119	0.750
27	0.375	58	0.438	89	0.250	120	1.000
28	0.438	59	0.625	90	0.438	121	0.500
29	0.750	60	0.375	91	0.438	122	0.313
30	0.500	61	0.625	92	0.313		
31	0.188	62	0.438	93	0.625		

*Compound NO. was the same to Table 1.

## Conclusion

In this study, a total of 122 kinds of volatile aroma components were detected by GC-MS analysis in 9 kinds of Fenghuang Dancong tea. Among all the components, 22 types of volatile aromas were found in all samples with the highest contributing components such as linalool, dehydrolinalool, β-linalool oxide I, linalool oxide II, geraniol, nerolidol, phytol and 2,2, 4,6,6-pentamethylheptane, etc. Similarity analysis revealed that a similarity ranging from 46.79% to 95.94% between the volatile aromas of the nine types of teas. Additionally, the nine types of Fenghuang dancong tea can be grouped into 4 categories when the abscissa distance is 10 according to the cluster analysis method. PCA result appeared that the cumulative variance contribution rate of the first five principal components showed 84.31%, indicating that the first three principal components are enough to explain all the variable information of the fragrance in Fenghuang Dancong tea. By using of the PCA method, our study proved that decane, octadecane, 2,2,4,6,6-pentamethylheptane, undecane, tetradecane, hexadecane, geraniol, dehydrolinalool, nerol and 1-octanol were the main source of floral and fruity aromas of Fenghuang Dancong Tea OPLS-DA result confirmed that 2,2,4,6,6-pentamethylheptane, dehydrolinalool, phenylacetaldehyde, nerolidol, linalool oxide I, hexanal, linalool oxide I, tetradecane, 3,4-dimethyl-o-phenylenediamine and benzaldehyde were the key differential compounds between the various types of tea samples.

## Supporting information

S1 Fig Overlaid GC-MS chromatographic of 9 type of Fenghuang Dancong tea. 1 Youhua xiang 2 Qilan xiang 3 Zhilan xiang 4 Yelai xiang 5 Moli xiang 6 Xinren xiang 7 Huangzhi xiang 8 Tongtian xiang 9 Qunti xiang.
(DOC)
