# Supplementary material for: Identification and similarity analysis of aroma substances in main types of Fenghuang Dancong tea
Source: PLoS One. 2020 Dec 21;15(12):e0244224. doi: 10.1371/journal.pone.0244224 (PMC7751878; doi:10.1371/journal.pone.0244224)
Supplement: S1 Fig — 1 Youhua xiang 2 Qilan xiang 3 Zhilan xiang 4 Yelai xiang 5 Moli xiang 6 Xinren xiang 7 Huangzhi xiang 8 Tongtian xiang 9 Qunti xiang. (DOC) [file pone.0244224.s001.doc]

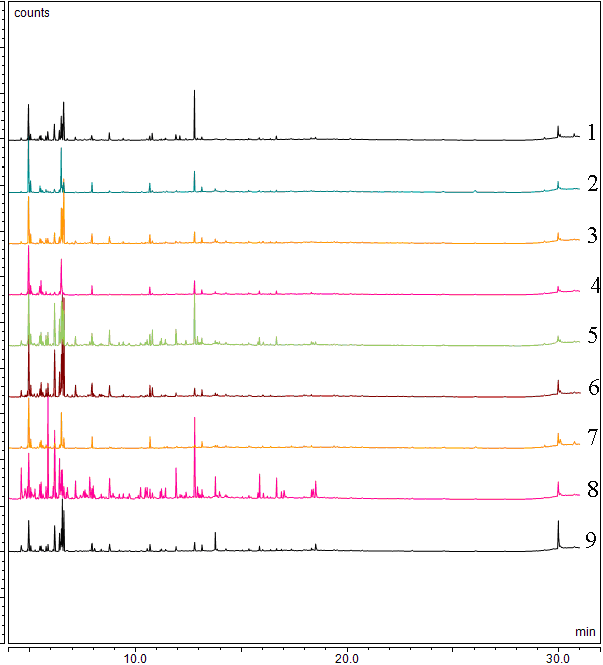


S1 Fig. Overlaid GC-MS chromatographic of 9 type of Fenghuang Dancong tea

1 Youhua xiang 2 Qilan xiang 3 Zhilan xiang 4 Yelai xiang 5 Moli xiang 6 Xinren xiang

7 Huangzhi xiang 8 Tongtian xiang 9 Qunti xiang
